# Phase I trial of isatuximab monotherapy in the treatment of refractory multiple myeloma

**DOI:** 10.1038/s41408-019-0198-4

**Published:** 2019-03-29

**Authors:** Thomas Martin, Stephen Strickland, Martha Glenn, Eric Charpentier, Hélène Guillemin, Karl Hsu, Joseph Mikhael

**Affiliations:** 1 University of California at San Francisco, San Francisco, CA USA; 2 Vanderbilt-Ingram Cancer Center, Nashville, TN USA; 3 Huntsman Cancer Institute, Salt Lake City, UT USA; 4 Sanofi, Cambridge, MA USA; 5 Sanofi, Vitry, France; 6 City of Hope Cancer Center and International Myeloma Foundation, Los Angeles, CA USA

**Keywords:** Myeloma, Cancer

## Abstract

This phase I dose-escalation/expansion study evaluated isatuximab (anti-CD38 monoclonal antibody) monotherapy in patients with relapsed/refractory multiple myeloma (RRMM). Patients progressing on or after standard therapy received intravenous isatuximab (weekly [QW] or every 2 weeks [Q2W]). The primary objective was to determine the maximum tolerated dose (MTD) of isatuximab. Overall, 84 patients received ≥ 1 dose of isatuximab. The MTD was not reached; no cumulative adverse reactions were noted. The most frequent adverse events were infusion reactions (IRs), occurring in 37/73 patients (51%) following introduction of mandatory prophylaxis. IRs were mostly grade 1/2, occurred predominantly during Cycle 1, and led to treatment discontinuation in two patients. CD38 receptor occupancy reached a plateau of 80% with isatuximab 20 mg/kg (highest dose tested) and was associated with clinical response. In patients receiving isatuximab ≥ 10 mg/kg, overall response rate (ORR) was 23.8% (15/63), including one complete response. In high-risk patients treated with isatuximab 10 mg/kg (QW or Q2W), ORR was 16.7% (3/18). Median (range) duration of response at doses ≥ 10 mg/kg was 25 (8–30) weeks among high-risk patients versus 36 (6–85) weeks for other patients. In conclusion, isatuximab demonstrated a manageable safety profile and clinical activity in patients with RRMM.

## Introduction

Over the past two decades, the development of novel chemotherapeutics, specific kinase inhibitors, and monoclonal antibodies (mAbs) has changed the treatment landscape for patients with hematologic malignancies^1,2^. In multiple myeloma (MM), agents including proteasome inhibitors (e.g., bortezomib, carfilzomib) and immunomodulatory drugs (e.g., lenalidomide, pomalidomide) have improved survival outcomes compared with previous cytotoxic regimens^3–6^. However, most patients with MM will still relapse following treatment with these agents, and the prognosis is particularly poor for those with recurrent disease following proteasome inhibitor and immunomodulatory drug treatment^7^.

Positive clinical data have recently emerged for mAbs directed against surface antigens on malignant plasma cells^8,9^. Both the anti-SLAMF7 mAb elotuzumab and the anti-CD38 mAb daratumumab received US Food and Drug Administration approval in 2015. These antibodies exert their cytotoxic effects through complement-dependent cellular cytotoxicity, antibody-dependent cellular cytotoxicity, and/or antibody-dependent cellular phagocytosis^10^. Elotuzumab demonstrated potent anti-MM activity in combination with lenalidomide and dexamethasone in patients with early relapsed/refractory MM (RRMM), yet showed no objective response as a single-agent^11^. In contrast, daratumumab received accelerated approval based on results from a phase II monotherapy and single-arm study showing an overall response rate (ORR) of 29.2% and median duration of response (DOR) of 7.4 months in heavily pretreated patients with RRMM^12^. Subsequently, two randomized phase III trials have demonstrated improved anti-MM activity when combining daratumumab with standard doublet therapies: bortezomib plus dexamethasone (CASTOR study: daratumumab/bortezomib/dexamethasone versus bortezomib/dexamethasone^13^) and lenalidomide plus dexamethasone (POLLUX study: daratumumab/lenalidomide/dexamethasone versus lenalidomide/dexamethasone^14^) in patients progressing after 1–3 prior lines of therapy. Both phase III studies showed marked improvements in progression-free survival (PFS) and ORR with triplet therapy (daratumumab/bortezomib/dexamethasone versus bortezomib/dexamethasone: hazard ratio for progression or death = 0.39; ORR 82.9% versus 63.2%, respectively; daratumumab/lenalidomide/dexamethasone versus lenalidomide/dexamethasone: hazard ratio for progression = 0.37; ORR 92.9% versus 76.4%, respectively). A phase Ib study of daratumumab in combination with pomalidomide and dexamethasone has also shown potent activity (ORR 59.2%) in patients who had received ≥ 2 prior lines of therapy, and these studies led to full US Food and Drug Administration approval for daratumumab as monotherapy and in these combinations for RRMM^15^.

Isatuximab is a novel immunoglobulin G1 kappa anti-CD38 mAb that binds selectively to a specific epitope on CD38. Preclinical studies suggest that isatuximab can target tumor cells through a combination of mechanisms, including antibody-dependent cellular cytotoxicity, antibody-dependent cellular phagocytosis, complement-dependent cellular cytotoxicity, and immune cell depletion/inhibition of immunosuppressive cells^16,17^. However, isatuximab appears unique among anti-CD38 mAbs as it can also induce direct apoptosis without cross-linking^16^. Furthermore, isatuximab is a potent inhibitor of CD38 enzymatic activity, which can impact on Ca^2+^ signaling^16^. Single-agent isatuximab has demonstrated anti-tumor activity in xenograft models of non-Hodgkin’s lymphoma, MM, and acute lymphoid leukemia^16,18^. Based on these encouraging preclinical data, a phase I, first-in-human study was initiated to evaluate the safety, tolerability, pharmacokinetics, pharmacodynamics, and efficacy of isatuximab monotherapy in patients with RRMM (*n* = 84) and other hematologic malignancies (*n* = 5). Only patients with RRMM are included in this report.

## Methods

### Eligibility

Initially, patients with RRMM and other hematologic malignancies were included in the study; however, based on early clinical activity and high CD38 expression in MM, the protocol was amended during dose-escalation (at 10 mg/kg weekly [QW]) to enroll only patients with a confirmed diagnosis of RRMM who had progressed on/after standard therapy, including an immunomodulatory drug and a proteasome inhibitor. CD38 expression was also removed from entry criteria. Patients had confirmed diagnosis of MM and had progressed on or after standard therapy. Eligible patients were ≥ 18 years old, had good performance status (Karnofsky performance status ≥ 60), good baseline organ function (aspartate aminotransferase, alanine aminotransferase, alkaline phosphatase, and bilirubin ≤ 2.5 × upper limit of normal; serum creatinine ≤ 2 × upper limit of normal), and adequate bone marrow function (absolute neutrophil count ≥ 1.0 × 10^9^/l, platelet count ≥ 75 × 10^9^/l, and hemoglobin ≥ 9 g/dl). Key exclusion criteria included: prior treatment with anti-CD38-directed therapy; another concomitant or prior malignancy; active HIV, AIDS, or hepatitis B or C infection; known central nervous system disease; pregnancy or breast-feeding; or known intolerance to infused protein products.

### Study design and treatment

This was a phase I, multicenter, open-label, dose-escalation study of single-agent isatuximab conducted in the USA, Spain, and France. Isatuximab was administered intravenously every 2 weeks (Q2W) or QW, in 2-week cycles, until disease progression, intolerable toxicity, or withdrawal of consent.

Isatuximab dose-escalation was planned from 0.0001 to 20 mg/kg (Supplementary Fig. S1). Following dose-escalation, two expansion cohorts (ECs) (EC1: standard-risk and high-risk patients; EC2: only high-risk patients) were added at 10 mg/kg Q2W. Another dosing cohort, evaluating the highest isatuximab dose (20 mg/kg QW), was added after efficacy and pharmacokinetic data became available from EC1. Premedication against infusion-related reactions (IRRs) was made mandatory from the 3 mg/kg Q2W cohort onward. Following the dose-escalation phases, two expansion cohorts (EC1: standard-risk and high-risk patients; EC2: only high-risk patients) of 18 patients each were added at 10 mg/kg Q2W. High-risk RRMM was defined as: abnormal genotype (del[17p], > 3 copies of 1q21, t[4;14], or t[14;16]) by cytogenetics or interphase fluorescence in situ hybridization; disease relapse within 6 months of autologous stem cell transplantation; or high-risk gene-expression profile (defined by investigator). For further details on dose-escalation and premedication see Supplementary Methods.

### Study objectives

The primary objective was to determine the maximum tolerated dose (MTD; highest dose at which dose-limiting toxicities [DLTs] occurred in < 2 of 6 patients, assessed during the first 4 weeks of treatment) of isatuximab. Secondary objectives were evaluation of safety/tolerability, pharmacokinetics/pharmacodynamics, and preliminary efficacy of isatuximab.

### Dose-limiting toxicities

A DLT was initially defined as an isatuximab-related occurrence of any of the following events: grade ≥ 3 non-hematologic toxicity; grade 4 neutropenia or grade 4 thrombocytopenia lasting > 5 days; grade ≥ 2 allergic reaction or hypersensitivity (i.e., infusion reactions [IRs]); or any other toxicity deemed by the investigators or sponsor to be dose-limiting. The DLT definition was amended at the 3 mg/kg Q2W cohort to eliminate grade ≤ 2 IR as part of the DLT definition, as patients experiencing a grade 2 IR before the end of the infusion were able to complete isatuximab dosing with appropriate management.

### Safety and efficacy assessments

Safety was evaluated continuously by physical examination, laboratory tests, and reports of adverse events (AEs) using National Cancer Institute Common Terminology Criteria for Adverse Events version 4.0. Isatuximab-related AEs beginning shortly after infusion were recorded as IRs and individual symptoms were recorded as AEs of special interest. Clinical responses were assessed according to the European Group for Blood and Marrow Transplant (EBMT) response criteria^19^. Disease responses were assessed every 28 days. Efficacy was assessed by ORR (at least partial response [PR]) and clinical benefit rate (CBR) (at least minimal response).

Treatment-emergent adverse events (TEAEs) were defined as adverse events (AEs) that developed, worsened (investigator’s judgment), or became serious during the on-treatment phase. IRs were considered adverse events of special interest and were followed closely. Additional safety assessments included: laboratory tests (hematology, serum chemistries, urinalyses, anti-drug antibodies), and pulmonary and cardiac evaluations.

Responses were assessed according to disease type. MM responses were classified according to the EBMT criteria^19^, with overall response rate (ORR) defined as attainment of at least partial response, and clinical benefit rate defined as attainment of at least minimal response; very good partial response is not included in the EBMT criteria.

### Pharmacokinetic/pharmacodynamic assessment

Isatuximab plasma concentrations were determined using a validated enzyme-linked immunoabsorption assay with a lower limit of quantification of 0.5 ng/ml. Individual pharmacokinetic parameters were estimated by non-compartmental analysis. Receptor occupancy (RO) was derived from receptor density assessed by a quantitative flow cytometry assay (see Supplementary Methods for further details).

### Statistical considerations

All analyses were performed on the all-treated population (patients who received ≥1 dose [even if incomplete] of isatuximab). Continuous data were summarized using descriptive statistics; categorical and ordinal data were summarized using number/percentage of patients. No statistical hypotheses were generated or power calculations performed. PFS (time from first dose to disease progression or death, whichever is first) was derived as a post-hoc variable and analyzed for patients treated at doses ≥ 10 mg/kg with the Kaplan–Meier method with patients from the EC2 cohort analyzed separately.

### Study oversight

The protocol was approved by ethics committees at each institution and the study was conducted in compliance with the Declaration of Helsinki and the International Conference on Harmonization guidelines. All patients provided written informed consent.

## Results

### Patient characteristics

Initially, patients with RRMM and other hematologic malignancies (three non-Hodgkin’s lymphoma, two chronic lymphocytic leukemia) were included in the study. Only patients with RRMM are included in the analysis presented here. Eighty-four patients with RRMM were treated between June 2010 and December 2014. Four RRMM patients were treated in the accelerated dose-escalation cohorts, 36 in the basic dose-escalation cohorts, 37 in the expansion-phase cohorts (EC1, *n* = 19; EC2, *n* = 18), and 7 in the 20 mg/kg QW cohort. The 0.3 and 3 mg/kg cohorts were each expanded to six patients due to DLTs consistent with IRs (see below), and three patients whose disease progressed before completion of Cycle 2, were replaced for DLT evaluation (in the absence of DLTs). All 84 patients were included in the all-treated population.

Patient demographics and select baseline disease characteristics are summarized in Table 1. Overall, patients had received a median (range) of 5 (1–13) prior lines of therapy, and 62% had received prior carfilzomib or pomalidomide. In the high-risk EC2 cohort, the median (range) number of prior lines was 5.5 (2–8), and 72% had received prior carfilzomib or pomalidomide. Eighty-nine percent of high-risk patients had abnormal cytogenetics (del[17p] [44%], 1q21 gain [56%], or t[4;14] [22%]).

**Table 1:** Baseline demographics and disease characteristics

Characteristics	All (*n* = 84)	Isatuximab dose		
		≤ 5 mg/kg (*n* = 21)	10 mg/kg (*n* = 49)	20 mg/kg (*n* = 14)
Age				
Median (range), years	64 (40–81)	64 (41–77)	62.9 (40–81)	63.5 (49–74)
≥ 65 years, *n* (%)	38 (45)	9 (43)	24 (49)	5 (36)
Male/female, *n* (%)	49 (58)/35 (42)	12 (57)/9 (43)	28 (57)/21 (43)	9 (64)/5 (36)
ECOG PS, *n* (%)				
0	11 (13)	1 (5)	8 (16)	2 (14)
1	58 (69)	15 (71)	32 (65)	11 (79)
2	15 (18)	5 (24)	9 (18)	1 (7)
Median time since diagnosis, years (range)	5.84 (1.2–22.8)	4.91 (1.8–9.9)	5.85 (1.2–22.8)	5.99 (3.0–12.9)
MM subtype, *n* (%)				
IgA	15 (18)	6 (29)	7 (14)	2 (14)
IgD	1 (1)	0	1 (2)	0
IgG	44 (52)	7 (33)	27 (55)	10 (71)
IgM	1 (1)	0	1 (2)	0
Light-chain (*κ* + *λ*)	23 (27)	8 (38)	13 (27)	2 (14)
ISS stage at baseline, *n* (%)				
I	29 (35)	9 (43)	15 (31)	5 (36)
II	30 (36)	7 (33)	19 (39)	4 (29)
III	23 (27)	4 (19)	14 (29)	5 (36)
Missing	2 (2)	1 (5)	1 (2)	0
Median BM PCs, % (range)	40 (0–100)	37 (0–90)	40 (0.8–100)	46 (0–85)
Albumin < 35 g/l, *n* (%)	31 (37)	7 (33)	22 (45)	2 (14)
B2M ≥ 5.5 mg/l, *n* (%)	23 (27)	4 (19)	14 (29)	5 (36)
EM plasmacytoma at baseline, *n* (%)	12 (14)	4 (19)	7 (14)	1 (7)
Median no. of prior treatment lines (range)	5 (1–13)	6 (2–13)	5 (1–13)	4.5 (2–7)
Prior SCT, *n* (%)	68 (81)	18 (86)	36 (73)	14 (100)
Prior treatments, *n* (%)				
Bortezomib	83 (99)	21 (100)	49 (100)	13 (93)
Carfilzomib	36 (43)	4 (19)	25 (51)	7 (50)
Lenalidomide	79 (94)	19 (90)	48 (98)	12 (86)
Pomalidomide	34 (40)	4 (19)	23 (47)	7 (50)
PI and IMiD	84 (100)	21 (100)	49 (100)	14 (100)

*B2M* β_2_ microglobulin, *BM* bone marrow, *ECOG PS* Eastern Cooperative Oncology Group performance status, *EM* extramedullary, *Ig* immunoglobulin, *IMiD* immunomodulatory drug, *ISS* International Staging System, *MM* multiple myeloma, *PC* plasma cell, *PI* proteasome inhibitor, *SCT* stem cell transplantation

Treatment regimen defined as ≥ 1 planned cycle of a single-agent or combination therapy, irrespective of whether therapy is given as part of a planned sequence

Treatment line defined as ≥ 1 planned cycle of single-agent or combination therapy, or a sequence of treatments in a planned manner^20^

### Treatment and disposition

All patients have discontinued treatment due to disease progression (*n* = 72 [85.7%]), other reasons (including patient preference) (*n* = 8 [9.5%]), or AEs (*n* = 4 [4.8%]).

Overall, the median (range) duration of isatuximab exposure was 11 (2–120) weeks, with a median of 5 (1–56) 2-week cycles administered. In patients treated at 10 and 20 mg/kg, the median duration of exposure was 14.4 weeks and 14.9 weeks, respectively. Median relative dose intensity of isatuximab was 98% and was consistent among dose levels. The median infusion time for the first and subsequent infusions was 3.50 and 2.60 h, respectively, for isatuximab 10 mg/kg, and 5.76 and 4.0 h, respectively, for isatuximab 20 mg/kg.

### Safety

**Fig. 1:**
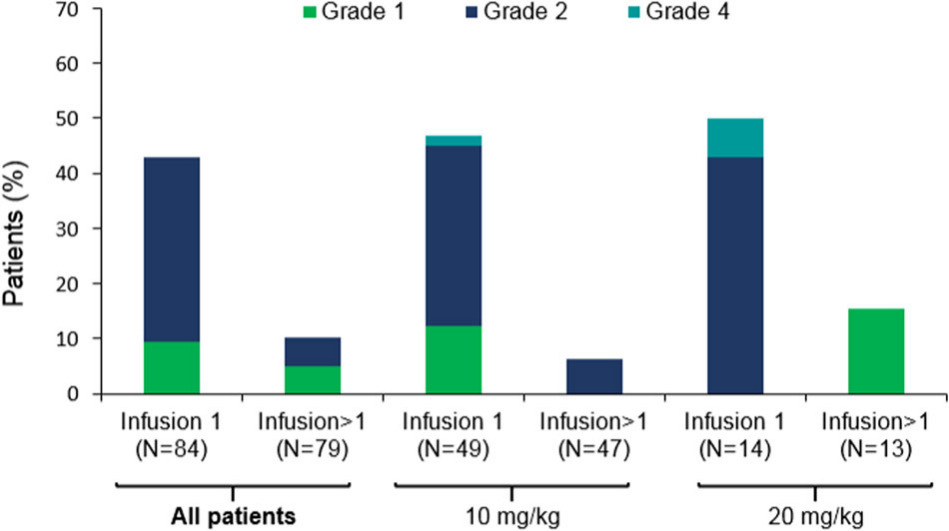
**Infusion reactions according to number of infusions and dose level**

The MTD was not reached. DLTs were observed in two patients during Cycle 1 (one each in the 0.3 and 3 mg/kg cohorts). Both were grade 2 IRs that were part of the original DLT definition (see Supplementary Information). Both patients completed the first infusion, did not discontinue therapy due to the IRs, and did not experience IRs in subsequent cycles. Following these events, the protocol was amended to remove grade ≥ 2 IRs from the DLT definition, as these events were not dose-dependent and had no sequelae. The protocol was also modified at this point to mandate premedication for IR prophylaxis. After the introduction of mandatory prophylactic treatment, 36/73 patients (49.3%) experienced AEs consistent with IRs. Of the patients (treated at the ≤ 0.3 mg/kg doses) who received the first dose of isatuximab before the institution of mandatory prophylaxis, IRs occurred in 3/7 patients (43%). Overall, IRs that were reported as AEs of special interest were grade 1/2 in 94% of patients (Fig. 1). At the isatuximab doses ≥ 10 mg/kg, 47.6% of patients experienced IRs with the first infusion, and 8.3% with subsequent infusions. IRs tended to resolve the same day either spontaneously or with treatment. The most common symptoms ( ≥ 5%) reported during IRs were chills, dyspnea (12% each), nausea (11%), headache (8%), chest discomfort (7%), and pyrexia (6%), all of which were grade 1/2 in intensity. Two patients discontinued treatment due to grade 4 IRs, one at 20 mg/kg Q2W (grade 4 apnea, definitely related to diphenhydramine and possibly related to isatuximab), the other at 10 mg/kg Q2W (grade 4 hypertension). Isatuximab infusions were interrupted in 29.8% of patients; the most common AE resulting in dose interruption was IR (27.0%). Only three patients had dose interruptions in subsequent infusions (all at 10 mg/kg).

Overall, the most common (> 10%) TEAEs, excluding IRRs and hematological TEAEs, were fatigue (37%), nausea (32%), upper respiratory tract infection (24%), and cough (23%) (Table 2). Grade 3/4 isatuximab-related TEAEs were reported in 17% of patients (Supplementary Table S1). Serious TEAEs were reported in 43% of patients. The most frequent grade 3/4 hematologic laboratory abnormalities during treatment were lymphopenia (34%), anemia (20%), thrombocytopenia (17%), and neutropenia (12%); the frequency of these abnormalities did not appear to be dose-dependent (Table 2). Grade 3/4 liver and renal abnormalities (laboratory assessment) occurred as follows: elevated aspartate aminotransferase, 4%; elevated alanine aminotransferase, 4%; elevated alkaline phosphatase, 1%; elevated creatinine, 5%.

**Table 2:** Most common^a^ TEAEs (regardless of relationship with study treatment)

TEAE	All patients (*n* = 84), *n* (%)		Isatuximab dose All grades/grade 3/4, no. of patients		
	All grades	Grade 3/4	≤ 5 mg/kg (*n* = 21)	10 mg/kg (*n* = 49)	20 mg/kg (*n* = 14)
Any TEAE	83 (99)	49 (58)	21/13	49/26	13/9
Most common TEAEs					
Fatigue	31 (37)	3 (4)	10/0	16/1	5/2
Nausea	27 (32)	0	6/0	19/0	2/0
Cough	19 (23)	0	5/0	11/0	3/0
URTI	20 (24)	0	3/0	13/0	4/0
Back pain	17 (20)	3 (4)	2/0	14/2	1/1
Diarrhea	17 (20)	0	3/0	11/0	3/0
Vomiting	14 (17)	0	2/0	10/0	2/0
Dyspnea	16 (19)	1 (1)	3/0	10/1	3/0
Headache	15 (18)	1 (1)	6/1	6/0	3/0
Pyrexia	16 (19)	2 (2)	6/1	9/1	1/0
Bone pain	12 (14)	3 (4)	4/1	8/2	0
Decreased appetite	12 (14)	0	1/0	9/0	2/0
Chills	11 (13)	0	5/0	6/0	0
Pneumonia	6 (7)	6 (7)	2/1	5/5	0/0
Laboratory abnormalities ^a,b^					
Anemia	80 (98)	16 (20)	20/4	48/11	12/1
Lymphopenia	65 (79)	28 (34)	15/8	40/18	10/2
Leukopenia	63 (77)	7 (9)	16/1	38/6	9/0
Thrombocytopenia	53 (64)	14 (17)	11/5	33/6	9/3
Neutropenia	37 (45)	10 (12)	7/1	25/9	5/0
AST increased	36 (43)	3 (4)	7/0	22/3	7/0
ALT increased	24 (29)	3 (4)	4/0	15/3	5/0
ALP increased	16 (19)	1 (1)	3/0	10/1	3/0
Creatinine increased	48 (58)	4/5	9/2	31/2	8/0

*ALP* alkaline phosphatase, *ALT* alanine aminotransferase, *AST* aspartate aminotransferase, *TEAE* treatment-emergent adverse event, *URTI* upper respiratory tract infection ^a^Adverse events occurring in ≥ 10% of patients (all grades) or > 5% (grade 3/4), excluding infusion reactions

^b^For laboratory abnormalities, percentages are calculated based on the number of evaluable patients for each parameter

Dose delay due to an AE occurred in 24 patients, most frequently due to infection (*n* = 14). Three patients experienced > 5 days of infusion delay due to an isatuximab-related AE: grade 3 neutropenia at Cycle 2 (10 mg/kg Q2W), two episodes of grade 2 upper respiratory tract infection for the same patient at Cycles 16 and 20 (10 mg/kg QW), and grade 3 pneumonia at Cycle 17 (3 mg/kg). Four patients discontinued treatment due to TEAEs: two patients with IRs described above, and one patient each due to grade 2 bone pain (5 mg/kg; not isatuximab-related), and fatal renal failure (10 mg/kg Q2W; not isatuximab-related). There were 11 other deaths, all occurring > 30 days after the last isatuximab dose and attributed to progressive disease (*n* = 10) or to reasons not related to isatuximab (bacterial meningitis/sepsis, *n* = 1).

### Pharmacokinetics and pharmacodynamics

In the accelerated dose-escalation cohorts, isatuximab was not detectable ( < 0.5 ng/ml). At higher doses, isatuximab pharmacokinetics showed moderate to high total variability (coefficient of variation 14–81% of isatuximab exposure) (Supplementary Fig. S2). Isatuximab pharmacokinetics appeared to be nonlinear, as exposure (area under the plasma concentration–time curve [AUC] _2 weeks_) increased in a greater than dose-proportional manner up to 20 mg/kg Q2W (Table 3). These data suggest the presence of target-mediated drug disposition. Some accumulation was observed following dosing at 10 or 20 mg/kg QW or Q2W, with accumulation highest at 20 mg/kg QW (at Cycle 3 AUC_1 week_ the accumulation ratio [geometric mean] was 1.7 and 2.6 for 10 mg/kg QW and 20 mg/kg QW, respectively) (Supplementary Table S2).

**Table 3:** Isatuximab plasma pharmacokinetic parameters at Cycle 1

Parameters	Isatuximab dose and schedule							
	0.3 mg/kg Q2W	1 mg/kg Q2W	3 mg/kg Q2W	5 mg/kg Q2W	10 mg/kg QW	10 mg/kg Q2W	20 mg/kg QW	20 mg/kg Q2W
No. of patients with evaluable PK	6	3	4	2	3	20	6	3
Infusion duration, *h*	2.53	4.38	4.53	4.28	2.30	2.32	4.88	5.88
*t*_max_, *h*	2.49	4.35	6.99	7.65	2.25	4.75	4.30	5.87
*C*_max_, μg/ml	2.09 (31)	13.5 (45)	55.3 (28)	135	183 (20)	180 (40)	356 (29)	469 (28)
AUC_last_, μg h/ml	16.5 (73)	674^a^	3120 (14)	14 200	17 400 (23)^c^	22 200 (50)	32 200 (33)^c^	49 900 (53)
AUC_1 week_, μg h/ml	16.5 (73)	460 (81)	3110 (14)	9 180	17 000 (22)	14 400 (44)	31 700 (31)	33 300 (46)
AUC_2 week_, μg h/ml	NC	NC	NC	13 100	NC	21 000 (54)^b^	NC	49 900 (53)

*AUC* area under the plasma concentration–time curve, *C*_last_ last measurable plasma concentration, *C*_max_ maximum plasma concentration, *NC* not calculated, *PK* pharmacokinetics, *Q2W* every 2 weeks, *t*_max_ time taken to reach *C*_max_

Data are mean (coefficient of variation %), except for infusion duration and *t*_max_, which are medians.

^a^*n* = 2

^b^*n* = 5

^c^*n* = 18

**Fig. 2:**
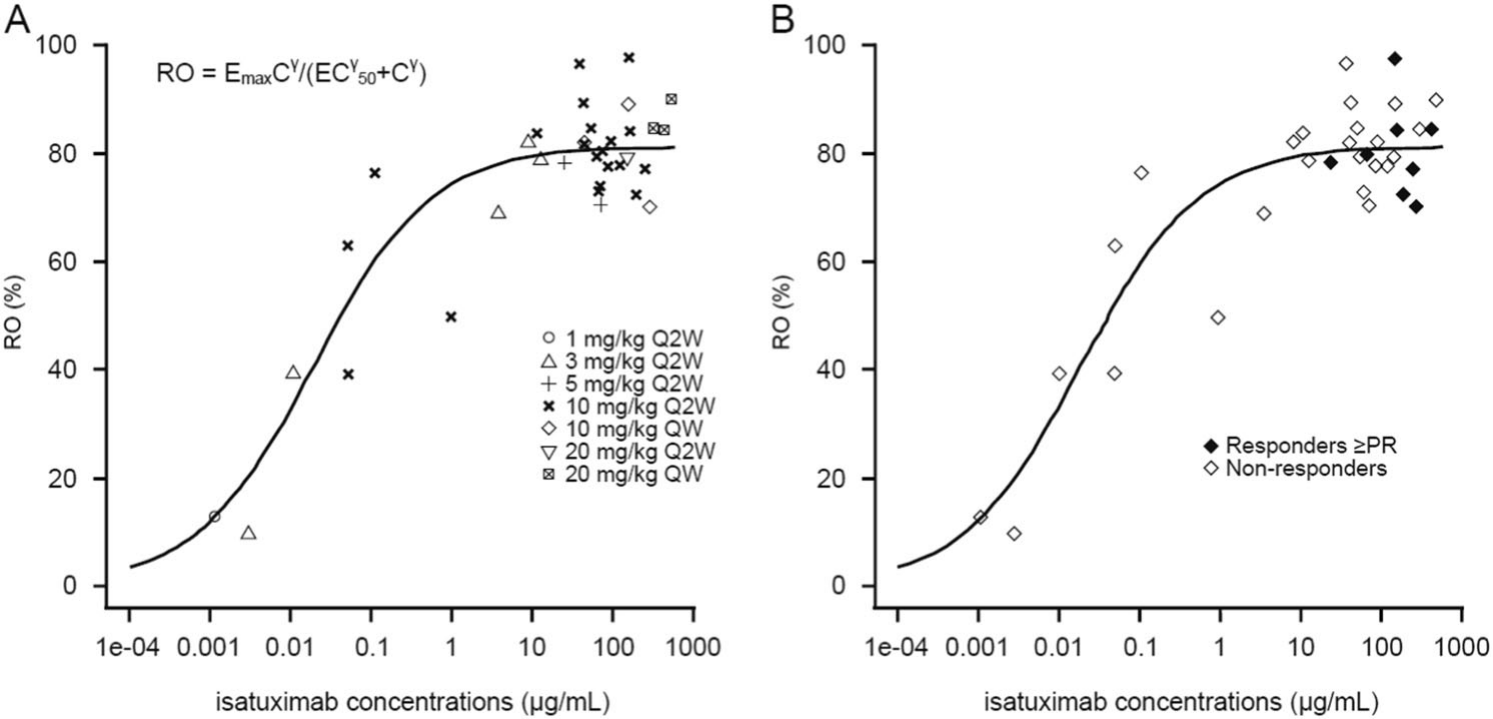
Relationship between receptor occupancy (RO), isatuximab concentration, and response. Relationship between **a** RO and isatuximab concentration and **b** RO, isatuximab concentration, and response. *E*_max_ = 81.3%, EC_50_ = 0.019 µg/ml, *γ* = 0.595. *C* isatuximab concentration, *EC*_50_ half maximal effective concentration, *E*_max_ maximum effect, *PR* partial response, *QW* every week, *Q2W* every 2 weeks, *RO* receptor occupancy

The relationship between isatuximab plasma concentration and RO at the end of Cycle 2 is described by the maximum effect attributable to the drug (*E*_max_) model (Fig. 2a), with an *E*_max_ of approximately 80%. The plateau for RO (i.e., *E*_max_) was consistently reached for concentrations corresponding to 20 mg/kg Q2W. RO varied at < 20 mg/kg and ranged from approximately 40 to 80% at 10 mg/kg Q2W. Notably, RO was ≥ 70% in patients who achieved a PR or better (Fig. 2b). With the association between isatuximab concentration and RO established, and pharmacokinetic exposure highest and least variable at 20 mg/kg, the 20 mg/kg QW cohort was added.

### Efficacy

**Fig. 3:**
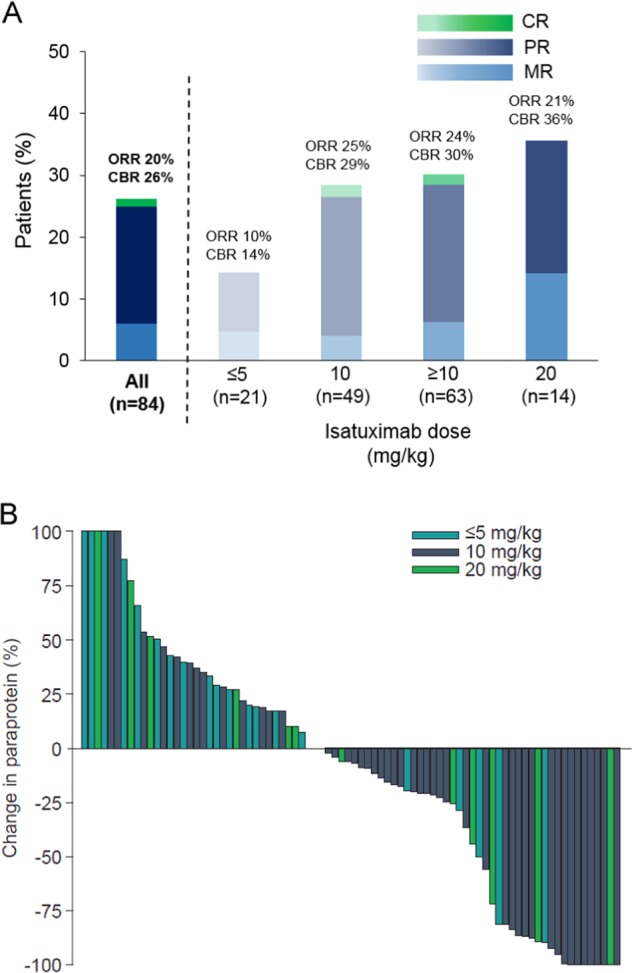
Summary of response data in patients with relapsed/refractory multiple myeloma. **a** Response histogram by isatuximab dose level, using European Group for Blood and Marrow Transplant criteria; **b** Waterfall plot of paraprotein change by isatuximab dose level in patients treated at isatuximab ≥ 1 mg/kg. Response not evaluable in three patients (1 at 10 mg/kg and 2 at 20 mg/kg). *CBR* clinical benefit rate, *CR* complete response, *MR* minimal response, *ORR* overall response rate, *PR* partial response

Objective responses were observed at doses ≥ 1 mg/kg (i.e., when RO became detectable). In patients treated with 1–5 mg/kg (*n* = 11) the ORR was 18.2% and the CBR was 27.3%. In patients treated with ≥ 10 mg/kg (*n* = 63), the ORR was 23.8% and the CBR was 30.2% (Fig. 3a). A complete response (CR) was observed in 1 patient (10 mg/kg QW)—a 76-year-old man with lambda light-chain disease who had received four prior lines of therapy; his best response to the last two lines, including alkylating agents and bortezomib, was disease progression. Disease assessment showed PR at Cycles 2 and 4, then CR until last disease assessment at Cycle 42. In the high-risk cohort, the ORR was 16.7% and CBR 27.8% (PR recorded in 3/16 patients with adverse cytogenetics, including one patient with high-risk gene-expression profiling). In patients with extramedullary disease, ORR was 25% (3/12 patients). A waterfall plot of M-protein changes is shown in Fig. 3b; 11 patients attained an M-protein decrease of > 90%. For patients treated at doses ≥ 10 mg/kg (excluding EC2 cohort), median PFS was 3.7 months (95% CI 2.56 to 5.78) (Table 4). For patients treated at 10 mg/kg Q2W in the EC2 cohort median PFS was 2.9 (95% CI 1.87 to 5.49) (Table 4).

**Table 4:** Progression-free survival—Kaplan–Meier estimates

	≥10 mg/kg (excluding EC2) (*N* = 45)	HR cohort (EC2) (*N* = 18)
Number (%) of events	33 (73.3)	16 (88.9)
Number (%) of patients censored	12 (26.7)	2 (11.1)
Kaplan–Meier estimates 25th quantile (95th CI) (months)	2.1 (1.12–2.89)	1.6 (0.85–2.79)
Median (95% CI) (months)	3.7 (2.56–5.78)	2.9 (1.87–5.49)
75th quantile (95th CI) (months)	9.2 (5.06–16.33)	5.9 (2.99–12.71)
Probability of surviving (95% CI)		
2 months	0.760 (0.630–0.890)	0.611 (0.386–0.836)
4 Months	0.471 (0.313–0.628)	0.438 (0.205–0.670)
6 Months	0.342 (0.185–0.499)	0.250 (0.042–0.458)
8 Months	0.308 (0.153–0.463)	0.125 (0.000–0.286)
12 Months	0.137 (0.015–0.258)	0.125 (0.000–0.286)
18 Months	0.068 (0.000–0.181)	0.063 (0.000–0.181)

In patients responding to isatuximab ≥ 10 mg/kg, median (range) time to first response was 4.29 (3.9–48.0) weeks. For patients treated at 10 mg/kg outside the high-risk cohort (*n* = 31), median (range) DOR ( ≥ PR) was 36.14 (6.1–85.3) weeks. Median (range) DOR in patients who received 10 mg/kg in the high-risk cohort was 25.29 (8.0–30.0) weeks.

## Discussion

This phase I study demonstrated that isatuximab monotherapy was generally well tolerated up to 20 mg/kg in patients with RRMM. IRs were the most common treatment-related AEs; these were grade 1/2 in 95% of patients, and occurred predominantly with the first infusion. Infusion interruption due to IRs occurred in 27% of patients, and only two patients discontinued treatment due to IRs.

Assessment of pharmacokinetics demonstrated that isatuximab exposure was nonlinear, as a result of target-mediated drug disposition. The initial pharmacokinetic/pharmacodynamic results from the dose-escalation cohorts and the first EC demonstrated the importance of RO, as only patients who attained RO ≥ 70% achieved a clinical response. As the plateau for RO was not consistently reached at doses up to 10 mg/kg, an additional dose cohort at 20 mg/kg QW was included. Overall, the pharmacokinetic parameters, including RO, were more favorable at this dose.

Isatuximab monotherapy demonstrated notable clinical activity with an ORR of 24% at isatuximab doses ≥ 10 mg/kg in this heavily pretreated RRMM population. Specifically, patients in this study had received a median of five prior lines of therapy, and over 60% had received pomalidomide or carfilzomib. Although there were no clear response differences observed between the 10 and 20 mg/kg dosing cohorts, the pharmacokinetic analyses suggest that the 20 mg/kg dose provides more consistent target saturation, especially in patients with bulky disease. Thus, the 20 mg/kg dose level has been further evaluated in subsequent monotherapy trials. Overall, the responses were rapid and durable, with a median time to first response of 4 weeks and a median DOR of approximately 25 weeks. An improvement in response quality from PR to CR was observed in one patient, although it should be noted that responses were assessed according to EBMT rather than International Myeloma Working Group criteria^20^, such that very good PR, an intermediate between PR and CR, was not assessed. In 2010, when this trial was initiated, the EBMT criteria were commonly used in MM clinical trials^21,22^.

This phase I study also reports promising results in patients with high-risk disease, as defined by cytogenetics or gene-expression profiling, suggesting that isatuximab therapy or immunotherapy is agnostic to previously defined adverse prognostic features. Clinical data for other mAb therapies, including elotuzumab^11^ and daratumumab^12^, further confirm that this therapeutic class is risk agnostic. The responses observed in this high-risk population were durable and notable because patients with high-risk disease generally have a shorter DOR^23^. However, only a small number of patients were included in the high-risk expansion cohort, thus further investigations are warranted, including the use of isatuximab in combination with other anti-MM therapies in patients with high-risk disease.

**Fig. 4:**
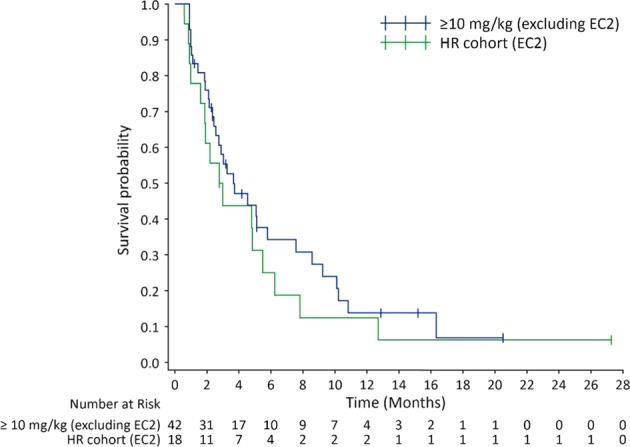
**Progression-free survival Kaplan–Meier plot**

The response rates observed in this study are similar to those observed with other single-agent therapies approved for use in RRMM, such as carfilzomib, pomalidomide, and daratumumab^12,24–26^. For example, results from the phase II daratumumab monotherapy study in heavily pretreated patients with advanced MM are comparable with those reported here; in the daratumumab 16 mg/kg arm, median ORR was 29% and median DOR was 32.2 weeks, compared with 24% and 25 weeks, respectively, at isatuximab doses ≥ 10 mg/kg (including the EC2 cohort)^12^. The median PFS for patients treated at doses ≥ 10 mg/kg (excluding the EC2 cohort) was also consistent with what was reported for daratumumab (3.7 months). As expected, the median PFS for patients treated at 10 mg/kg Q2W in the dedicated HR risk cohort was shorter (Fig. 4). The overall incidence of IRRs with isatuximab monotherapy (47.2%) appears higher than the incidence of IRRs reported in a phase II study of daratumumab monotherapy (42%)^12^, although grade 3/4 IRRs occurred less frequently with isatuximab (grade 3, 0; grade 4, 2.2%) than with daratumumab (grade 3, 5%; grade 4, 0). Prophylactic therapies to prevent IRs are mandatory for both agents, with the difference that post-infusion medications are required to reduce the risk of delayed IRs with daratumumab.

Emerging data suggest that mAbs are most effective when used in combination with other therapies^10^. Elotuzumab monotherapy showed no objective response^27^, but potent activity with lenalidomide/dexamethasone^11^. The recent phase III data from the POLLUX (daratumumab/lenalidomide/dexamethasone^14^) and CASTOR (daratumumab/bortezomib/dexamethasone^13^) studies suggest an even greater benefit with using daratumumab in these combinations in early disease relapse (first to third relapse). The addition of isatuximab to lenalidomide/dexamethasone has shown clinical promise in heavily pretreated patients with RRMM^28^. There have been no studies that have attempted to investigate the optimum sequencing of antibody therapies, and whether particular agents show improved efficacy with certain combinations or lines of therapy remains unclear.

Three isatuximab phase III studies are currently ongoing; one evaluating isatuximab together with pomalidomide and dexamethasone in patients who have received at least 2 prior lines, a second combining isatuximab with carfilzomib and dexamethasone in patients who have received 1–3 prior lines, and a third study evaluating isatuximab in combination with bortezomib, lenalidomide, and dexamethasone in patients with newly diagnosed MM who are ineligible for transplant. Additional studies are under way for both isatuximab and daratumumab, as well as for elotuzumab, evaluating various combinations in front-line, relapsed disease, and smoldering MM. Whether one agent will emerge as superior in a particular treatment combination or setting is yet to be seen. With regard to CD38 antibodies, isatuximab and daratumumab bind to different and unique epitopes on CD38, and in vitro studies reveal differences in their mode of action, most notably that isatuximab promotes apoptosis without cross-linking^16^. Whether these differences will translate into differences in clinical activity, or whether patients who are refractory to one anti-CD38 antibody will respond to another, is yet unknown.

In conclusion, isatuximab monotherapy was generally well tolerated and demonstrated preliminary efficacy in the treatment of RRMM. Although an MTD was not reached, the optimum monotherapy dose/schedule was selected as 20 mg/kg weekly for four doses followed by Q2W dosing. An ongoing study is evaluating isatuximab monotherapy and includes patients who have failed to respond to prior daratumumab treatment (NCT02514668). Additional studies are planned that will evaluate isatuximab in combination with other immuno-oncology drugs, including checkpoint blockers. CD38 receptors are present on T-regulatory cells and other immunosuppressive cells, and emerging data suggest that CD38 therapy can stimulate cytotoxic T-cell responses^29^. The unique binding differences between the CD38 antibodies may, perhaps, prove most important when these agents are combined with other immuno-oncology agents. Further studies are necessary to identify the combination with the highest benefit/risk profile to patients with RRMM. Phase III studies are evaluating isatuximab (10 mg/kg) in combination with pomalidomide and dexamethasone, as well as with carfilzomib and dexamethasone in RRMM. Additional studies are ongoing to help identify the optimal combination and/or clinical setting in which to use isatuximab therapy.

## Supplementary information

Supplementary Material

Supplemental Fig. S1 Study design (online only)

Supplemental Fig. S2 Individual isatuximab plasma pharmacokinetic profiles following the first intravenous infusion of isatuximab (online only)
