# Supplementary material for: Phase I trial of isatuximab monotherapy in the treatment of refractory multiple myeloma
Source: Blood Cancer J. 2019 Mar 29;9(4):41. doi: 10.1038/s41408-019-0198-4 (PMC6440961; doi:10.1038/s41408-019-0198-4)
Supplement: Supplementary file 1 — Supplementary Material [file 41408_2019_198_MOESM1_ESM.docx]

**Supplementary information**

**Methods**

**Study design and treatment**

Two dose-escalation schemes were used (Supplementary Fig. S1): an accelerated dosing schedule for the first five dose levels (DLs) (0.0001–0.1 mg/kg once every 2 weeks [Q2W]; one patient per DL) and a basic, 3+3-based design for DLs 0.3–5 mg/kg Q2W. The starting dose was selected based on the minimal anticipated biological effect level approach and in vitro receptor occupancy in relevant animal species, and in the absence of an acceptable surrogate antibody for toxicological assessment. For the 10 and 20 mg/kg DLs, 6 patients were to be treated even in the absence of a dose-limiting toxicity (DLT; defined below).

**Premedications**

The protocol was also amended at the 3 mg/kg Q2W cohort to mandate the use of standard premedications including methylprednisolone 100 mg intravenously (IV) (or equivalent), diphenhydramine 25–50 mg orally or IV (or equivalent), famotidine 20 mg IV (or equivalent), and acetaminophen 650–1000 mg orally, 30–60 minutes before each isatuximab infusion.

**Pharmacokinetic/pharmacodynamic assessment**

Isatuximab plasma concentrations were determined using a validated enzyme-linked immunoabsorption assay with a lower limit of quantification of 0.5 ng/ml. Individual pharmacokinetic parameters were calculated after the first and third isatuximab administrations with noncompartmental analysis using WinNonlin software version 5.2.1 (Certara USA, Inc., Princeton, NJ, USA). Receptor occupancy (RO) was determined by a quantitative flow cytometry assay. Isatuximab plasma concentration and RO at the end of Cycle 2 were fitted using a maximum effect (E_max_) equation with Hill coefficient (γ):

RO=E_max_C^γ^ /(EC_50_^γ^ + C^γ^).

Where C is the isatuximab concentration and EC_50_ is half maximal effective concentration.

**Pharmacokinetic sampling**

With Q2W dosing in the dose-escalation phase, blood samples were collected before, during, and after isatuximab administration on Days 1, 2, 3, and 8 of Cycle 1, then on Day 1 (before drug administration) of subsequent cycles, and at the end of study. With weekly dosing, blood samples were collected before, during, and after administration on Days 1, 2, 3, 4, and 8 of Cycles 1 and 3, and on Days 1 and 8 (before drug administration) of Cycle 2 and all other cycles.

**Statistical considerations**

The pharmacokinetic population comprised patients in the all-treated population with an evaluable pharmacokinetic parameter. Continuous data for the all-treated population were summarized using descriptive statistics. Categorical and ordinal data were summarized using number and percentage of patients at each DL. No formal statistical hypotheses were generated or power calculations performed.

**Reference**

1. Blade J, et al. Criteria for evaluating disease response and progression in patients with multiple myeloma treated by high-dose therapy and haemopoietic stem cell transplantation. Myeloma Subcommittee of the EBMT. European Group for Blood and Marrow Transplant. *Br J Haematol.* 1998; 102: 1115–1123.

**Supplemental Table S1** **Drug-related events occurring in >5% patients (online only)**

| TEAE | All patients (n=84), n (%) | | Isatuximab dose  All grades/grade 3/4, No. of patients | | |
| --- | --- | --- | --- | --- | --- |
|  | All grades | Grade 3/4 | ISA ≤5 mg/kg (n=21) | ISA 10 mg/kg (n=49) | ISA 20 mg/kg (n=14) |
| Any TEAE | 63 (75) | 14 (17) | 15/2 | 38/11 | 10/1 |
| Infusion reaction | 41 (49) | 2 (2) | 9/0 | 24/1 | 8/1 |
| Nausea | 12 (14) | 0 | 3/0 | 8/0 | 1/0 |
| Chills | 11 (13) | 0 | 5/0 | 6/0 | 0 |
| Dyspnea | 11 (13) | 0 | 2/0 | 7/0 | 2/0 |
| Fatigue | 9 (11) | 0 | 4/0 | 5/0 | 0 |
| Headache | 9 (11) | 0 | 5/0 | 2/0 | 2/0 |
| Pyrexia | 7 (8) | 1 (1) | 4/1 | 2/0 | 1/0 |
| Chest discomfort | 6 (7) | 0 | 0 | 4/0 | 2/0 |
| URTI | 5 (6) | 0 | 2/0 | 2/0 | 1/0 |

TEAE treatment-emergent adverse event, URTI upper respiratory tract infection.

**Supplemental Table S2** **Isatuximab plasma pharmacokinetic parameters at Cycles 1 and 3 with weekly (QW) dosing (online only)**

|  | **Isatuximab dose and schedule** | | | |
| --- | --- | --- | --- | --- |
| **Parameter** | **10 mg/kg QW**  **Cycle 1** | **10 mg/kg QW**  **Cycle 3** | **20 mg/kg QW**  **Cycle 1** | **20 mg/kg QW**  **Cycle 3** |
| No. of patients included/total | 3/6 | 4/5 | 6/7 | 6/6 |
| Infusion duration, h | 2.30 | 2.60 | 4.88 | 4.07 |
| t_max_, h | 2.25 | 4.30 | 6.83 | 8.07 |
| C_max_, µg/ml | 183 (20) | 326 (67) | 356 (29) | 737 (27) |
| AUC_last_, µg·h/ml | 17 400 (23) | 40 600 (61) | 32 200 (33)^a^ | 85 800 (43)^a^ |
| AUC_1week_, µg·h/ml | 17 000 (22) [16 700] | 37 100 (66) [28 500] | 31 700 (31)^a^ [30 400] | 86 600 (43)^a^ [30 400] |

AUC area under the plasma concentration–time curve, C_last_ last measurable plasma concentration, C_max_ maximum plasma concentration, t_max_ time taken to reach C_max_

Data are mean (coefficient of variation %), except for infusion duration and t_max_, which are medians. Geometric mean data are also shown in square brackets for AUC_1week_

^a^n=5
