# Supplementary material for: Phase I trial of isatuximab monotherapy in the treatment of refractory multiple myeloma
Source: Blood Cancer J. 2019 Mar 29;9(4):41. doi: 10.1038/s41408-019-0198-4 (PMC6440961; doi:10.1038/s41408-019-0198-4)
Supplement: Supplementary file 2 — Supplemental Fig. S1 Study design (online only) [file 41408_2019_198_MOESM2_ESM.docx]

**Supplemental Fig. S1** Study design (online only)


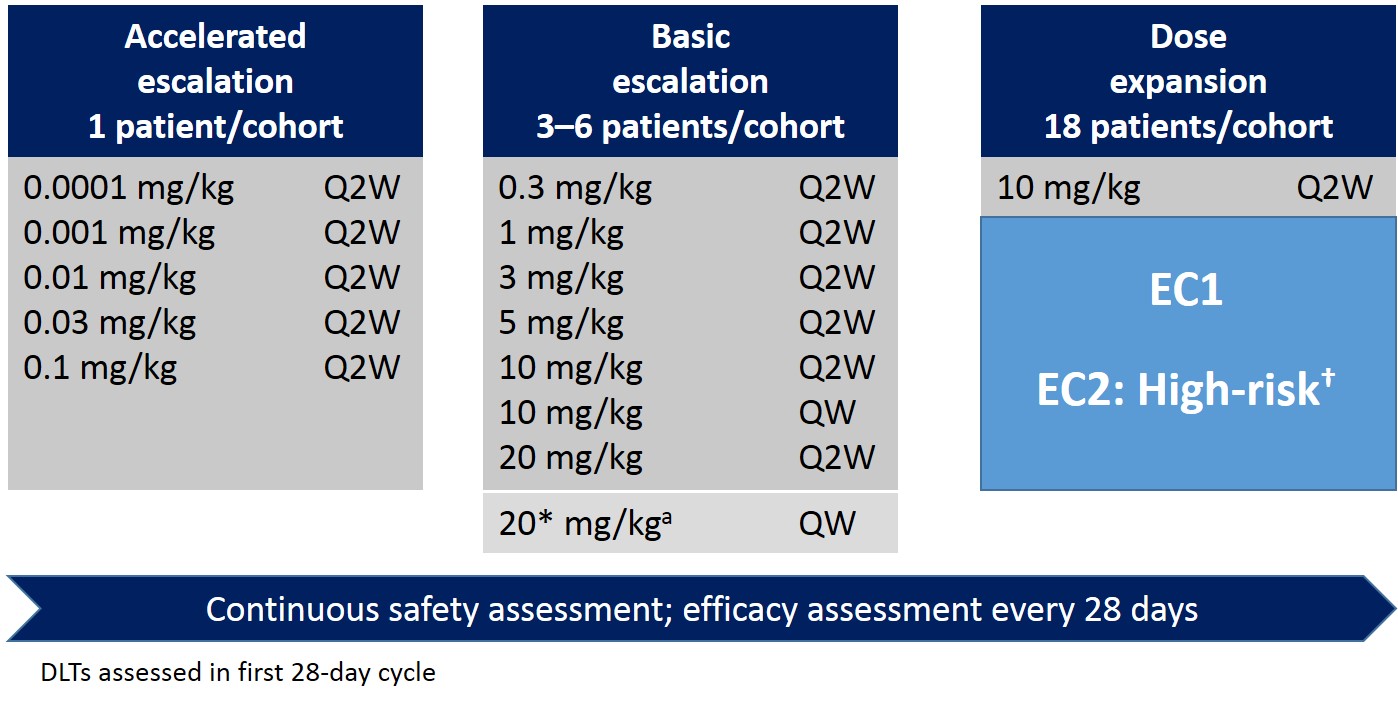


DLT dose-limiting toxicity, EC expansion cohort, MM multiple myeloma, QW every week, Q2W every 2 weeks

^a^Cohort testing 20 mg/kg QW added after expansion cohorts, based on pharmacokinetic data from the expansion cohorts

^†^High-risk MM defined as: del(17p), t(4;14), t(14;16), t(14;20), or >3 copies of 1q21; relapse <6 months after autologous stem cell transplant; high-risk gene-expression profile.
