# Supplementary material for: Phase I trial of isatuximab monotherapy in the treatment of refractory multiple myeloma
Source: Blood Cancer J. 2019 Mar 29;9(4):41. doi: 10.1038/s41408-019-0198-4 (PMC6440961; doi:10.1038/s41408-019-0198-4)
Supplement: Supplementary file 3 — Supplemental Fig. S2 Individual isatuximab plasma pharmacokinetic profiles following the first intravenous infusion of isatuximab (online only) [file 41408_2019_198_MOESM3_ESM.docx]

**Supplemental Fig. S2** Individual isatuximab plasma pharmacokinetic profiles following the first intravenous infusion of isatuximab (online only)


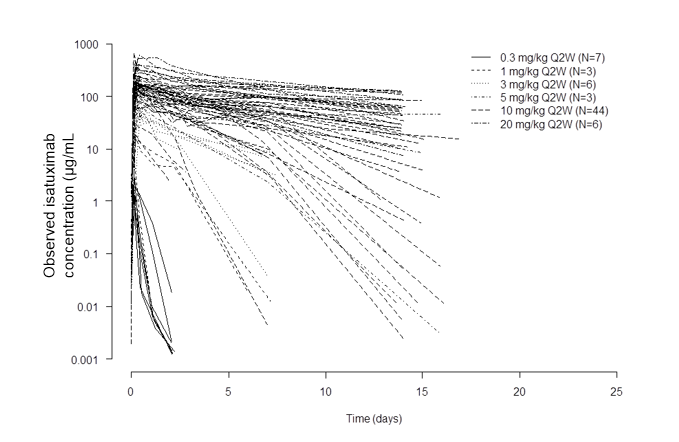


*Q2W* once every 2 weeks
